# Fully-automated global and segmental strain analysis of DENSE cardiovascular magnetic resonance using deep learning for segmentation and phase unwrapping

**DOI:** 10.1186/s12968-021-00712-9

**Published:** 2021-03-11

**Authors:** Sona Ghadimi, Daniel A. Auger, Xue Feng, Changyu Sun, Craig H. Meyer, Kenneth C. Bilchick, Jie Jane Cao, Andrew D. Scott, John N. Oshinski, Daniel B. Ennis, Frederick H. Epstein

**Affiliations:** 1 Department of Biomedical Engineering, University of Virginia, Health System, Box 800759, Charlottesville, VA 22908 USA; 2 Department of Medicine, University of Virginia Health System, Charlottesville, VA USA; 3 Department of Cardiology, St. Francis Hospital, New York, NY USA; 4 Cardiovascular Magnetic Resonance Unit, The Royal Brompton Hospital, London, United Kingdom; 5 Department of Radiology and Imaging Sciences, Emory University School of Medicine, Atlanta, GA USA; 6 Department of Radiology, Stanford University, Stanford, CA USA

**Keywords:** DENSE, Cardiac MRI, Machine learning, Deep learning, Phase unwrapping, Strain analysis, Segmental strain, Global strain, Heart

## Abstract

**Background:**

Cardiovascular magnetic resonance (CMR) cine displacement encoding with stimulated echoes (DENSE) measures heart motion by encoding myocardial displacement into the signal phase, facilitating high accuracy and reproducibility of global and segmental myocardial strain and providing benefits in clinical performance. While conventional methods for strain analysis of DENSE images are faster than those for myocardial tagging, they still require manual user assistance. The present study developed and evaluated deep learning methods for fully-automatic DENSE strain analysis.

**Methods:**

Convolutional neural networks (CNNs) were developed and trained to (a) identify the left-ventricular (LV) epicardial and endocardial borders, (b) identify the anterior right-ventricular (RV)-LV insertion point, and (c) perform phase unwrapping. Subsequent conventional automatic steps were employed to compute strain. The networks were trained using 12,415 short-axis DENSE images from 45 healthy subjects and 19 heart disease patients and were tested using 10,510 images from 25 healthy subjects and 19 patients. Each individual CNN was evaluated, and the end-to-end fully-automatic deep learning pipeline was compared to conventional user-assisted DENSE analysis using linear correlation and Bland Altman analysis of circumferential strain.

**Results:**

LV myocardial segmentation U-Nets achieved a DICE similarity coefficient of 0.87 ± 0.04, a Hausdorff distance of 2.7 ± 1.0 pixels, and a mean surface distance of 0.41 ± 0.29 pixels in comparison with manual LV myocardial segmentation by an expert. The anterior RV-LV insertion point was detected within 1.38 ± 0.9 pixels compared to manually annotated data. The phase-unwrapping U-Net had similar or lower mean squared error vs. ground-truth data compared to the conventional path-following method for images with typical signal-to-noise ratio (SNR) or low SNR (p < 0.05), respectively. Bland–Altman analyses showed biases of 0.00 ± 0.03 and limits of agreement of − 0.04 to 0.05 or better for deep learning-based fully-automatic global and segmental end-systolic circumferential strain vs. conventional user-assisted methods.

**Conclusions:**

Deep learning enables fully-automatic global and segmental circumferential strain analysis of DENSE CMR providing excellent agreement with conventional user-assisted methods. Deep learning-based automatic strain analysis may facilitate greater clinical use of DENSE for the quantification of global and segmental strain in patients with cardiac disease.

## Background

Myocardial strain imaging is sensitive and prognostic for the assessment of heart disease, with potential advantages over imaging of left ventricular (LV) ejection fraction (LVEF) [1]. Among various strain imaging methods, cardiovascular magnetic resonance (CMR) cine displacement encoding with stimulated echoes (DENSE) [2–4] CMR uniquely measures heart motion by encoding myocardial displacement into the signal phase, which facilitates high measurement accuracy [5], high reproducibility of global and segmental strain [6, 7], and rapid computation of displacement and strain [5, 8]. These properties translate to benefits in clinical performance. For example, cine DENSE shows superiority over late gadolinium enhanced (LGE) CMR and feature tracking in predicting major adverse cardiac events after myocardial infarction [9] and predicting outcomes of heart failure patients treated with cardiac resynchronization therapy [10]. Cine DENSE also detects contractile dysfunction in childhood obesity [11] and adult type 2 diabetes even when LVEF is normal [12].

While low-rank [13, 14] and reduced field-of-view [15] methods have been developed recently to accelerate data acquisition for DENSE, there remains a need and opportunity to accelerate DENSE strain analysis and to eliminate all steps that require user assistance. Image analysis of cine DENSE to compute LV whole-slice (global) and segmental strain requires the following steps: (a) segmentation of the LV myocardium, (b) identification of the anterior right-ventricular (RV) insertion point into the LV to align the American Heart Association 16-segment model [16], (c) unwrapping of potentially wrapped displacement-encoded phase values of the myocardium, (d) computation of the spatiotemporal displacement field, and (e) computation of strain. Currently, LV segmentation of DENSE is typically performed using motion-guided segmentation [8], which requires manual segmentation of the LV epicardial and endocardial borders at a single cardiac phase, followed by automated propagation of these borders to all other phases (guided by the measured myocardial displacements). User intervention is sometimes needed to adjust the segmentation results. Identification of the anterior RV insertion point is currently performed manually by an experienced user. Also, phase unwrapping is typically performed using a path-following method [5], and this method requires user selection of seed points placed in regions known to not have phase wrapping. After segmentation, identification of the anterior RV insertion point, and phase unwrapping, the remaining steps to compute displacement and strain are performed automatically without user assistance, as described [4, 5, 17, 18].

In this study we aimed to develop a fully-automated post-processing approach for DENSE. Recently, deep learning (DL) methods, particularly convolutional neural networks (CNN), have shown promising results for segmentation and analysis of various CMR techniques [19–29]. In this work, we developed a pipeline for fully-automated analysis of cine DENSE data using four CNNs to (a) identify the LV epicardial border, (b) identify the LV endocardial border, (c) identify the anterior RV-LV insertion point, and (d) perform phase unwrapping of the LV myocardium. The proposed pipeline eliminates all user intervention and reduces the time for image analysis. To validate the proposed approach, each new step was compared with expert-user or ground-truth methods and the end-to-end processing of global and segmental strains were compared to previously-validated user-assisted conventional DENSE analysis methods [17].

## Methods

### Dataset

For training and testing CNNs, cine DENSE data were collected from five medical centers (University of Virginia, Charlottesville, Virginia, USA, Emory University, Atlanta, Georgia, USA, Royal Brompton Hospital, London, United Kingdom, St. Francis Hospital, Roslyn, New York, USA, and Stanford University, Stanford, California, USA). This research was performed in accordance with the Declaration of Helsinki and in accordance with protocols approved by the institutional review boards of participating institutions. All participants provided written informed consent.

Short-axis cine DENSE CMR data from 38 heart-disease patients and 70 healthy subjects were used for network training and testing. Twenty-six datasets were acquired using 1.5 T systems (Magnetom Avanto or Aera, Siemens Healthineers, Erlangen, Germany) and 82 were acquired using 3 T systems (Magnetom Prisma, Skyra, or Trio, Siemens Healthineers). The types of heart disease included dilated cardiomyopathy, hypertrophic cardiomyopathy, coronary heart disease, hypertension, acute coronary syndrome and heart failure with left bundle branch block. For each subject, 1–5 short-axis slices were acquired, each with 20–59 cardiac phases. Training data included 12,415 short-axis DENSE images from 64 randomly selected subjects, and 20% of all training data were used for model validation. Forty-four datasets, including 25 healthy subjects and 19 patients imaged at both field strengths, were selected for the test data (10,510 total 2D images, including those with displacement encoded in both the x- and y-directions).

### Cine DENSE image acquisition protocol

Cine DENSE image acquisition parameters included a pixel size of 1.56 × 1.56 mm^2^–2.8 × 2.8 mm^2^, FOV = 200 mm^2^ (using outer volume suppression) to 360 mm^2^, slice thickness = 8 mm, a temporal resolution of 17 msec (with view sharing), 2D in-plane displacement encoding using the simple three-point method [30], displacement-encoding frequency = 0.1 cycles/mm, ramped flip angle with final flip angle of 15°, echo time = 1.26 − 1.9 msec, and a spiral k-space trajectory with 4–6 interleaves.

### Overview of image analysis pipeline

**Fig. 1:**
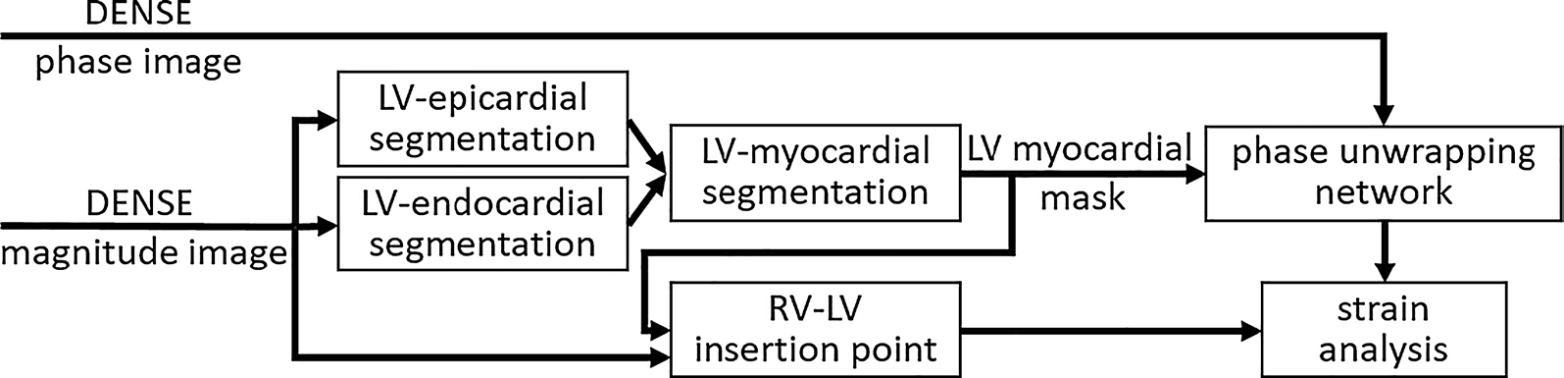
Diagram of fully-automated strain analysis pipeline for cine DENSE. *LV*  left ventricular, *RV*  right ventricular

We designed a fully-automatic DENSE analysis pipeline (Fig. 1) comprised of the following steps: (a) LV segmentation, (b) identification of the anterior RV-LV insertion point, (c) phase unwrapping, and (d) displacement and strain analysis. Steps (a)–(c) utilize CNNs, and step (d) uses previously-developed and validated fully-automatic methods [5, 31].

### CNN for LV segmentation

To create the ground-truth LV segmentation data, manual image annotation was performed for DENSE magnitude-reconstructed images. The LV endocardial and epicardial borders were manually traced for all frames using *DENSEanalysis* software [17]. To automatically segment the LV from DENSE magnitude images, we trained one U-Net to extract the epicardial border, and another to extract the endocardial border, and we identified the myocardial pixels by performing a logical XOR between the two masks. The 2D U-Net networks utilized the structure presented by Ronneberger [32] with modifications to get the best results for the proposed application. Specifically, in the contracting path, each encoding block contains two consecutive sets of dilated convolutional layers with filter size 3 × 3 and dilation rate 2, a batch normalization layer and a rectified linear activation layer. Compared with traditional convolutions, dilated convolutions can increase the receptive field size without increasing the number of parameters and showed improved performance in our experiments. Padding was used in each convolutional operation to maintain the spatial dimension. Between each encoding block, pooling layers with step size of 3 × 3 and stride 2 were applied to reduce the spatial dimension in all directions. The number of features was doubled for the next encoding block. Four symmetric encoding and decoding blocks were used in the contracting and expanding path, respectively. Each decoding block contained two consecutive sets of deconvolutional layers with filter size 3 × 3, a batch normalization layer and a rectified linear activation layer. The output of each encoding block in the contracting path was concatenated with those in the corresponding decoding block in the expanding path via skip-connections. The final segmentation map contained two classes: background and endocardium or epicardium. The loss function was the summation of the weighted pixel-wise cross entropy and soft Dice loss. The assigned class weights were 1 for background, 2 for endocardium in the endocardial network and 3 for the epicardial network. During training, data augmentation on-the-fly was performed by applying random translations, rotations and scaling followed by a b-spline-based deformation to the input images and to the corresponding ground-truth label maps at each iteration. This type of augmentation has the advantage that the model sees different data at each iteration. We used 400 epochs to train each network; therefore, each image was augmented 400 times. After applying the random transformations to the label maps, a threshold value of 0.5 was applied to the interpolated segmentation to convert back to binary values [33]. To improve the accuracy and smoothness of the segmented contours, during testing, each image was rotated 9 times at an interval of 40 degrees and the corresponding output probability maps were rotated back and averaged [34]. Hereafter, we refer to this testing process as “testing augmentation”.

### CNN to identify the anterior RV-LV insertion point

The anterior RV-LV insertion point is the location of the attachment of the anterior RV wall to the LV, and its location defines the alignment of the American Heart Association 16-segment model [16] typically used for segmental strain analysis of the LV. As the first frame of cine DENSE images generally has poor blood-myocardium contrast, we trained a U-Net to detect the anterior RV-LV insertion point on early-systolic frames (frames 5 and 6), where the insertion point is reliably well visualized. To create the ground-truth data, one expert user identified one point in these frames from magnitude-reconstructed DENSE images. During network training, instead of using that point as an absolute ground-truth, which only provides very limited information to the network to learn and suffers from severe class imbalance, we defined a circle with a six-pixel radius around that point as the network target. The network’s inputs were the DENSE magnitude image and the segmented LV binary mask obtained by the aforementioned myocardial segmentation networks as an additional input channel. The network’s output is the probability map of a circle for which the center of mass is defined to be the detected RV-LV insertion point. The same aforementioned U-Net structure was used. The loss function was the combination of the absolute difference and the soft Dice between the target and the output probability map computed using a Sigmoid function. The same on-the-fly data augmentation was applied during training, but testing augmentation was not used for this network.

### CNN for phase unwrapping

In phase-reconstructed CMR images, the phase value is inherently confined to the range (− π, π). However, in cardiac DENSE in order to balance displacement sensitivity, signal-to-noise ratio (SNR), and suppression of artifact-generating signals, displacement-encoding frequencies that lead to phase shifts of greater than π are typically used, and ± 1 cycle of phase wrapping typically occurs during systole [5]. Thus, phase unwrapping is required to convert phase to displacement.

The unwrapped phase ${\psi }_{ij}$ can be estimated from the potentially-wrapped measured phase ${\varphi }_{ij}$ as follows:

1 $${\psi }_{ij}={\varphi }_{ij}+2\pi {k}_{ij}$$

where ${k}_{ij}$ is an integer and where $-\pi {<\varphi }_{ij}<\pi$. The phase unwrapping problem requires determining ${k}_{ij}$ for each pixel indexed by *i* and *j*. Thus, the phase unwrapping can be defined as a semantic segmentation problem [35], and we pursued such an approach where the network would label each pixel as belonging to one of three classes (no wrap, -2π wrapped, or + 2π wrapped) as shown in Table 1.

**Table 1:** Phase unwrapping label definition and the corresponding classes

Class	${k}_{ij}$	Label
No wrap	0	0
Wrapped myocardium (− 2π)	− 1	1
Wrapped myocardium (+ 2π)	+ 1	2

To create the ground truth for unwrapped phase images, we used a highly accurate but very slow phase unwrapping method based on multiple phase prediction pathways and region growing [36]. We also visually checked the results of this method, frame by frame, and discarded all frames with unwrapping errors. The same dilated U-Net structure with three output classes was trained using a pixel-wise cross-entropy loss function. The network’s input was the segmented phase-reconstructed DENSE image and the output was the wrapping label map. With this design, after applying the CNN, the value of ${k}_{ij}$ is known for each pixel. Then by multiplying ${k}_{ij}$ by 2π and adding the result to the input wrapped image, the unwrapped phase is computed.

**Fig. 2:**
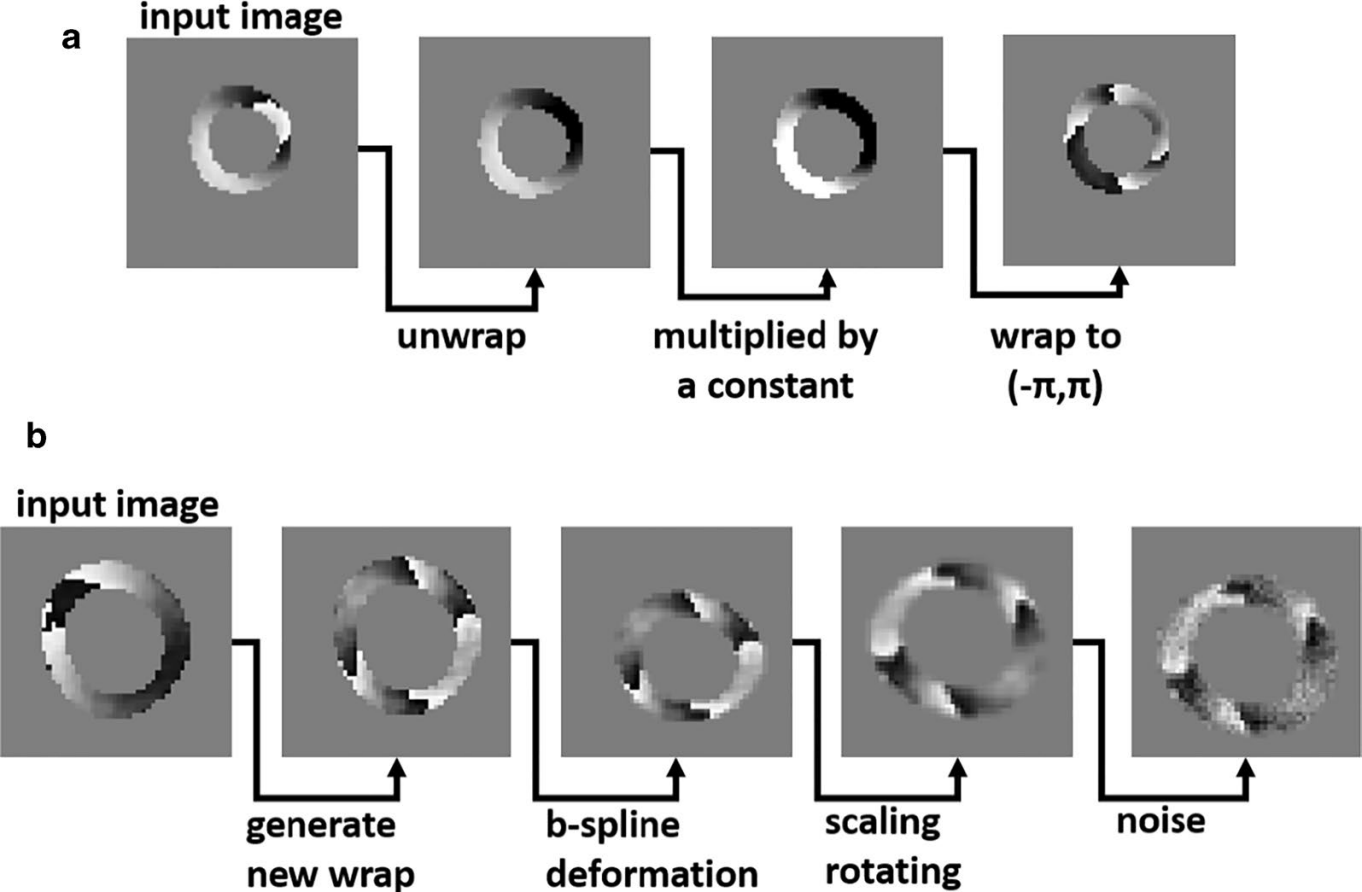
Data augmentation for the phase-unwrapping convolutional neural networks (CNN). Figure **a** shows how a new phase-wrapping pattern is generated during data augmentation using an original wrapped image as input, performing phase unwrapping, scaling of the unwrapped phase, and wrapping to the range of (− π,π). Figure **b** demonstrates an example of serial operations to generate augmented data

For this network, testing augmentation was not applied. However, we applied training augmentation by adding Gaussian noise with a mean of zero and a randomly chosen standard deviation between (0, 0.75) to simulate different signal-to-noise ratios and by manipulating the unwrapped ground truth data to generate new wrapped data. Data augmentation is the key point as overfitting is avoided and the network is trained on data with lower SNR and more wrapping patterns. To create augmented new wrapped data, an unwrapped ground-truth phase image is multiplied by a random constant number between 0.8 and 2.0, and then it is wrapped to the range (− π,π). For each augmented phase image, the ${k}_{ij}$ value is known and if it is 0, 1, or -1 then it is used for training. Figure 2a illustrates how a new phase-wrapping pattern is generated during augmentation and Fig. 2b demonstrates an example of how different operations can be applied to create augmented data. For this network, we randomly generated transformations comprised of translation, rotation, scaling, shearing, and b-spline deformation and applied them to the training images along with random phase manipulation and random noise. In total, 7 random augmentations were applied to each training image.

### Network training and testing

To obtain the best model performance, the final model of each network was trained using data from 64 subjects. Network training was performed on a Titan Xp GPU (NVIDIA Corporation, Santa Clara, California, USA) with 12 GB RAM over 400 epochs using an Adam optimizer at a learning rate of 5E-4 and a mini batch size of 10. The times to train the myocardial segmentation networks (endocardium and epicardium), RV-LV insertion point network, and phase unwrapping network were 34, 48, and 30 h, respectively. The networks were implemented using Python (version 3.5; Python Software Foundation, www.python.org) with the Tensorflow machine-learning framework (version 1.12.0) [37].

### Quantitative evaluation of the methods

To quantitatively evaluate the results of myocardial segmentation, the DICE similarity coefficient [38] was computed. This metric measures the overlap between the ground-truth segmentation (A) and the CNN’s segmentation (B) as follow:

2 $$DICE= \frac{2\times \left|A\cap B\right|}{\left|A\right|+\left|B\right|}$$

DICE coefficient is normalized between 0 and 1, where “0” indicates complete dissimilarity and “1” indicates complete agreement.

In addition, to measure the maximum and average distances between the myocardial ground-truth and the CNN-generated contours, the Hausdorff distance (${D}_{H}$) and the mean surface distance (MDS) were computed as follows. Given two sets of points $A=({a}_{1},\dots ,{a}_{n})$ and $B=({b}_{1},\dots ,{b}_{m})$, and an underlying distance $d(a,b)$ which is defined as the Euclidean distance $d\left(a,b\right)= \Vert a-b\Vert$, ${D}_{H}$ and MDS are given by:

3 $$\begin{aligned} D_{H} \left( {A,B} \right) = & \max \left( {h\left( {A,B} \right),h\left( {B,A} \right)} \right) \\ h\left( {A,B} \right) = & \mathop {\max }\limits_{a \in A} \mathop {(\min \left( {d\left( {a,b} \right)} \right)}\limits_{b \in B} \\ \end{aligned}$$

4 $$\begin{aligned} MSD = & mean\left( {h_{mean} \left( {A,B} \right),h_{mean} \left( {B,A} \right)} \right) \\ h_{mean} \left( {A,B} \right) = & \frac{1}{n}\mathop \sum \limits_{a \in A} \mathop {({\text{min}}\left( {d\left( {a,b} \right)} \right)}\limits_{b \in B} \\ \end{aligned}$$

To assess the accuracy of identifying the RV-LV insertion point position, the Euclidean distance between the expert-selected point and the centroid of the automatically-selected region was calculated.

To evaluate the phase-unwrapping CNN, we compared it with the widely-used path-following method [5] using mean squared error (MSE). The ground-truth unwrapped phase was computed using the phase-unwrapping method based on multiple phase prediction pathways and region growing [36].

For images with SNR typical of routine DENSE protocols [15, 39] (phase SNR of approximately 22), MSE referenced to ground truth were evaluated for the proposed U-Net and the path-following method. Similar to the phase SNR of velocity-encoded phase contrast imaging [40], we calculated the DENSE phase SNR as

$${ {phase SNR}}= \Vert \frac{{mean}\left({{unwrapped\, phase\, of\, end-systolic\, ROI}}\right)}{{stdev}\left({{phase\, of \,end-diastolic\, myocardium}}\right)}\Vert$$

where the mean unwrapped phase of an end-systolic region of interest (ROI) measures the DENSE phase in the region with greatest displacement (representing the signal of interest), and the standard deviation of the phase of the end-diastolic myocardium provides a measure of the standard deviation of phase at a cardiac frame where the mean phase is essentially zero. Because SNR can be lower than typical in some circumstances (such as when imaging patients with implanted devices), we also evaluated the two methods for lower SNR data generated by adding noise to our datasets. For low-SNR data, as we did not have true unwrapped-phase data to use as a ground-truth, we synthetically created the low-SNR data (with phase SNR = 5–10) from our test data by adding noise with zero mean and with standard deviation of 0.75. Adding noise to the original wrapped phase data could change the wrapping class of any image pixel. As the label of the pixel may not be the same as the corresponding pixel in the original data, for the low-SNR data we compared the U-Net with the path-following method by calculating the MSE between the unwrapped phase and the typical-SNR unwrapped ground truth.

To evaluate the full pipeline shown in Fig. 1 for global and segmental circumferential strain analysis of the LV, correlations and Bland–Altman analyses were performed comparing the proposed deep-learning based method and the conventional user-assisted semi-automated method (*DENSEAnalysis,* github open-source software, developed by us and others [17]). In *DENSEAnalysis*, a 10^th^-order polynomial was used for temporal fitting and a spatial smoothing parameter of 0.8 was selected.

## Results

### LV segmentation and anterior RV-LV insertion point

**Fig. 3:**
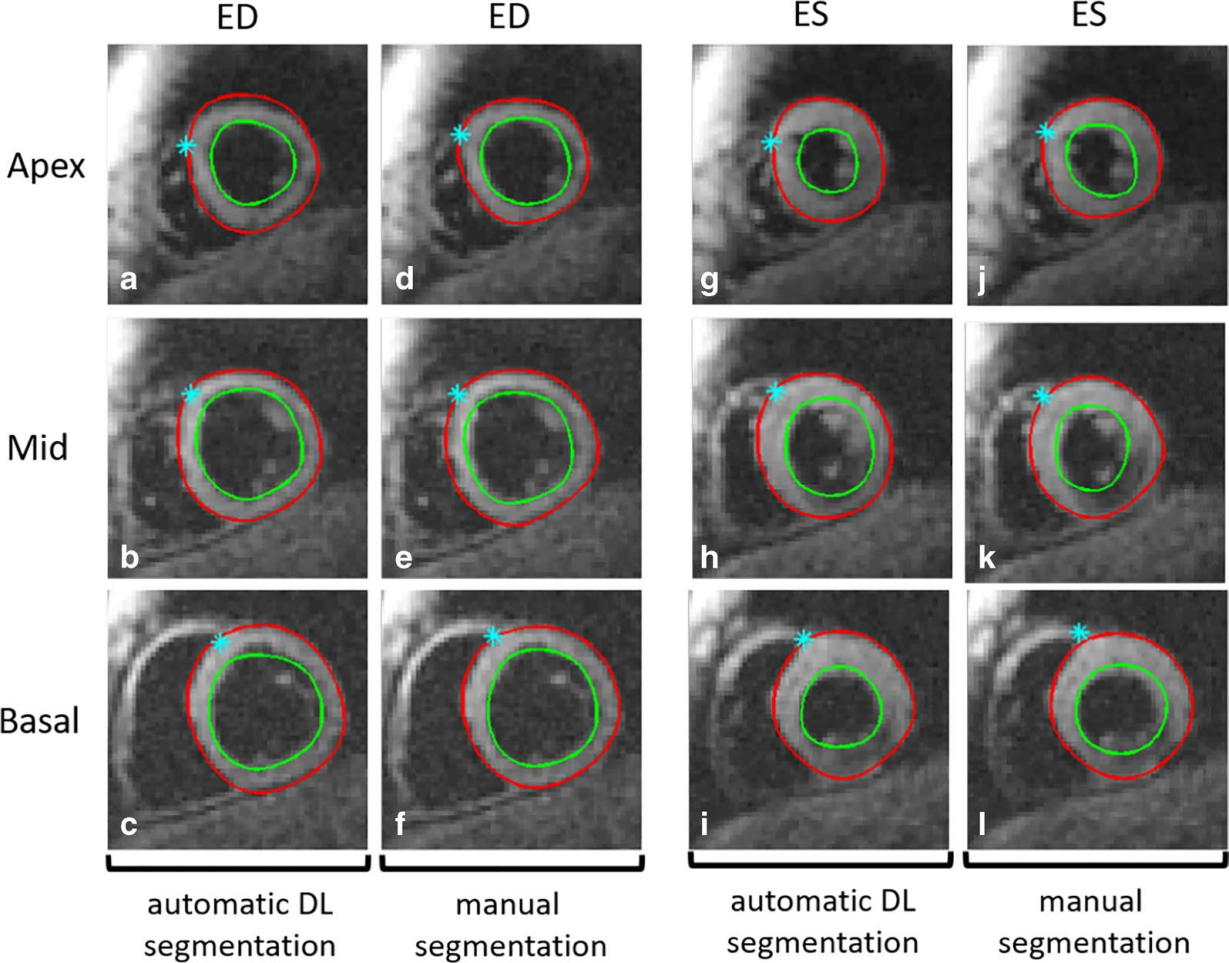
An example of automatic deep learning (DL) LV segmentation and identification of anterior RV-LV insertion points by U-Nets (**a**–**c**, **g**–**i**), and the corresponding results provided by an expert user (**d**–**f**, **j**–**l**). Results are shown at end diastole (ED) and end systole (ES). The epicardial contour is in red, the endocardial contour is in green, and the anterior RV-LV insertion point is depicted with a cyan asterisk

Evaluation of the U-Nets for LV segmentation using 5,255 test images resulted in a DICE coefficient of 0.87 ± 0.04, a Hausdorff distance of 2.7 ± 1 pixel (equivalent to 5.9 ± 2.2 mm), and a mean surface distance of 0.41 ± 0.29 pixels (0.9 ± 0.6 mm). The computation times for determining the epicardial and endocardial contours for a single DENSE image, including test augmentation, were 0.16 ± 0.02 s, 0.15 ± 0.01 s, respectively. The typical semi-automatic LV segmentation time for DENSE is 3–5 min for all cardiac phases, which corresponds to about 6 s per frame. The RV-LV insertion point was detected within 1.4 ± 0.9 pixels compared to the manually annotated data. The computation time for detecting the RV-LV insertion point was 2.4 ± 0.2 s for all cardiac phases. An expert reader uses approximately 20 s to manually define the point. Figure 3 shows examples of the automatically and manually segmented LV epicardial and endocardial contours and the identification of the anterior RV-LV insertion point on short axis images at end-diastolic and end-systolic frames.

### Phase unwrapping

**Fig. 4:**
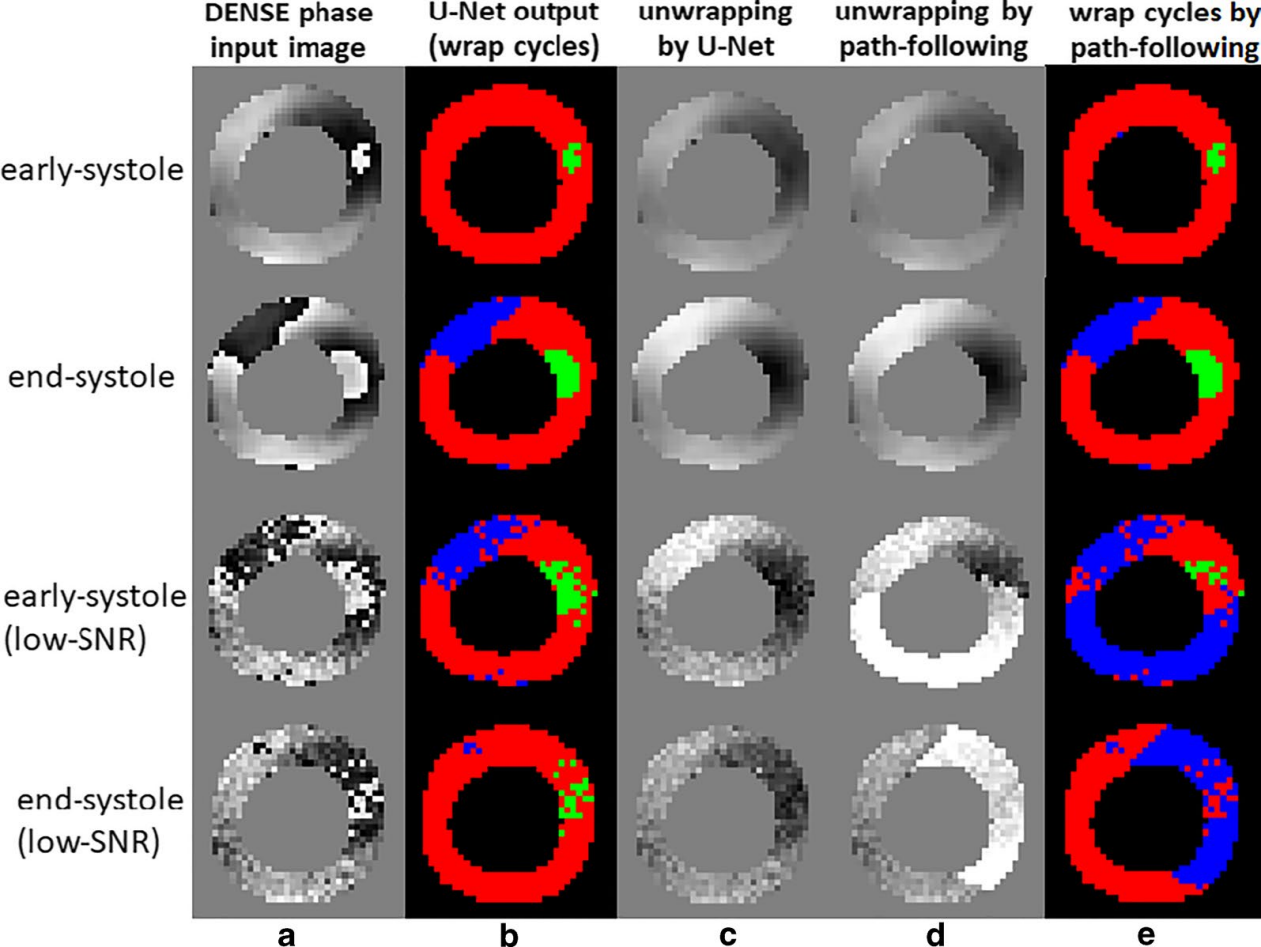
Demonstration of phase unwrapping of DENSE images (column **a**) using a semantic-segmentation U-Net, and comparison with the path-following method. For DENSE images with typical SNR (top two rows), the semantic segmentation U-Net correctly identified wrapped pixels (column **b**). Both the U-Net and the path-following methods performed phase unwrapping without errors (top two rows, columns **c**, **d**). For low-SNR data (bottom two rows), the U-Net successfully performed semantic segmentation and phase unwrapping, however the path-following method led to large phase-unwrapping errors, which are also depicted in wrap cycle maps (**e**)

The phase-unwrapping U-Net performed well on both typical-SNR and low-SNR DENSE phase images. The MSE values for the semantic-segmentation U-Net and the standard path-following method are provided in Table 2. MSE was similar for typical-SNR data using the U-Net and conventional path following, and was lower for low-SNR data using the U-Net (p < 0.05). The time for DL phase unwrapping for all cardiac phases was 3.5 ± 0.2 s, which was similar to path following method of 3.5 ± 0.7 s. Figure 4 illustrates an example where the U-Net and the path-following method were both successful for typical-SNR data and where the semantic-segmentation U-Net outperformed the path-following method for low-SNR data.

**Table 2:** Comparison of a semantic-segmentation U-Net and the path-following method for phase-unwrapping of displacement encoding with stimulated echoes (DENSE) images of the heart

		MSE
Typical SNR	U-Net	0.1 ± 0.2
	Path-following	0.08 ± 0.08
Low SNR	U-Net	0.07 ± 0.09*
	Path-following	0.22 ± 0.49

Mean squared error (MSE) values are reported for DENSE images with typical signal-to-noise ratio (SNR) and low-SNR

^*^Indicates p < 0.05

### Strain analysis

**Fig. 5:**
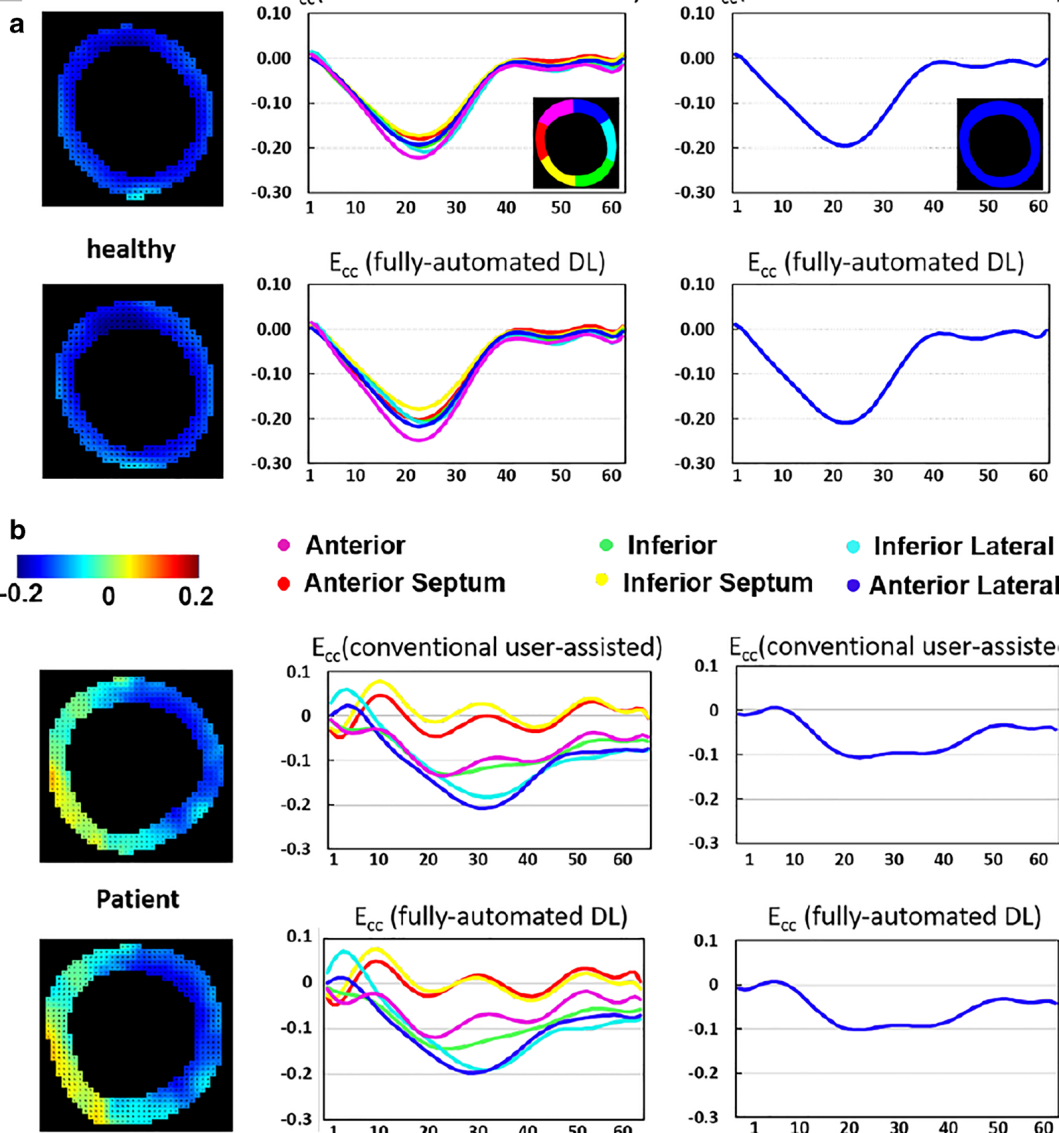
Example end-systolic circumferential strain (E_cc_) maps (left column) and segmental (middle column) and global (right column) circumferential strain–time curves from a healthy subjects (**a**) and a heart failure patient (**b**) are shown. For these example mid-ventricular slices, close agreement is seen when comparing the DL-based fully-automatic method and the conventional user-assisted method

**Fig. 6:**
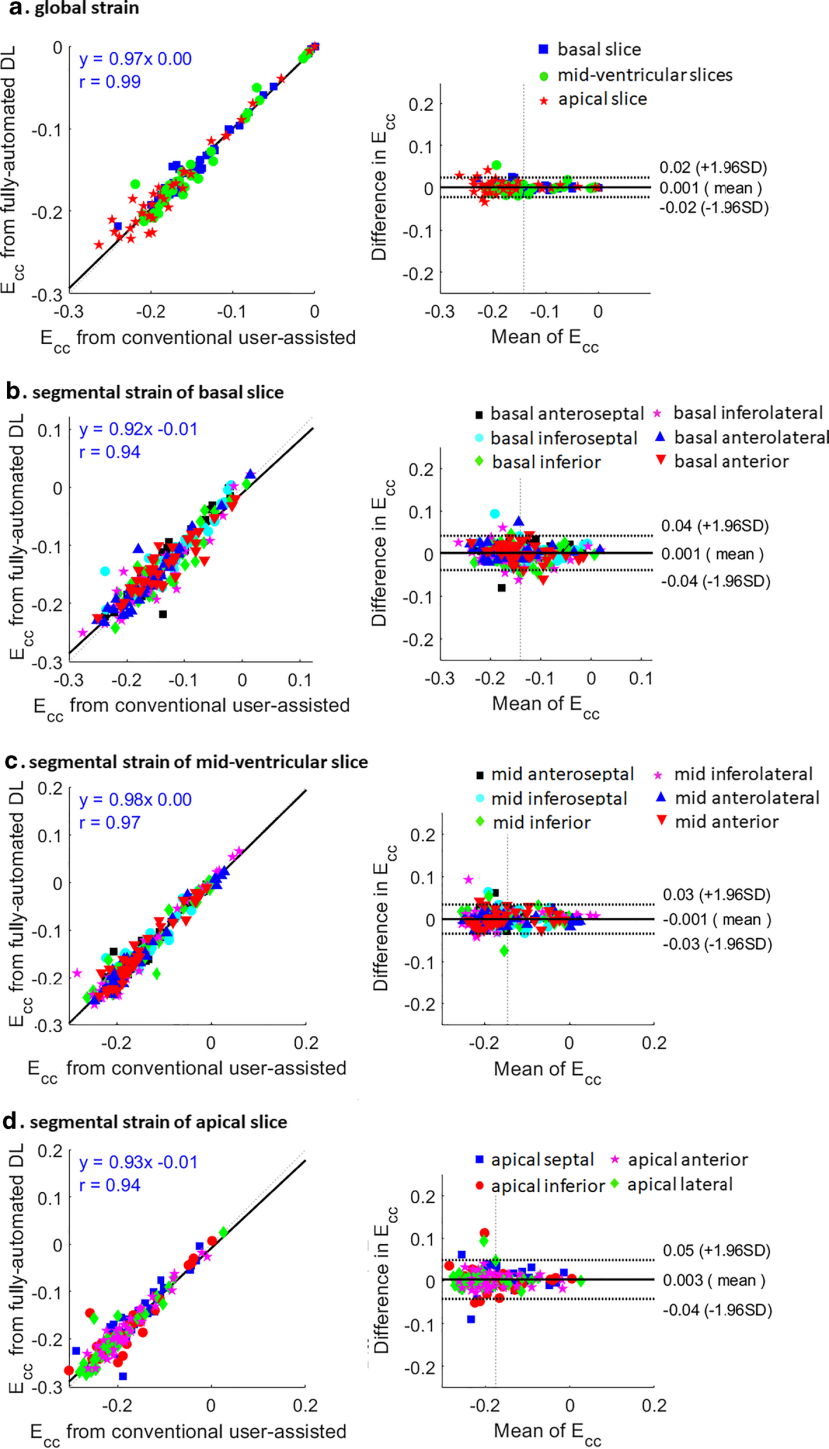
Correlation (left) and Bland–Altman (right) plots for global (**a**) and segmental (**b**–**d**) circumferential strains at end systole of basal (**b**), mid-ventricular (**c**), and apical slices (**d**) computed using the conventional user-assisted and the fully-automated DL methods

We used the fully-automated DL methods to compute global and segmental circumferential strain for all test data and compared the results with the conventional user-assisted DENSE analysis methods [17]. Figure 5a and b show two examples of end-systolic strain maps, global and segmental strain–time curves computed using the DL-based automated methods and the conventional method for a healthy subject and a HF patient with a septal strain defect. Very close agreement between the DL-based and conventional DENSE analysis methods is seen in Fig. 5. Figure 6a shows the Bland–Altman plot and the linear correlation comparing the DL and conventional DENSE analysis methods for end-systolic global circumferential strain. The bias was 0.001 and the limits of agreement were − 0.02 and 0.02. For the linear correlation, r = 0.97 and the slope was 0.99. A slice-by-slice analysis of segmental strain is provided in Fig. 6b–d, and shows very good agreement of segmental end-systolic strain between the fully-automated DL method and the conventional method. The biases were 0.00 ± 0.03 and the limits of agreement were − 0.04 to 0.04 for basal segments, − 0.03 to 0.03 for mid-ventricular segments, and − 0.04 to 0.05 for apical segments. Excellent correlations (r = 0.94–0.97, slope = 0.92–0.98) were found for all segments of all slices.

Table 3 shows the mean ± SD of segmental circumferential strain and the variance ± SD within each segment at end-systole for the mid-ventricular slice of all test data. Two-way ANOVA showed that while there are differences between segments for both mean circumferential strain (p < 0.05) and variance of circumferential strain (p < 0.05), there are no significant differences between the conventional user-assisted and DL-based fully-automatic methods for mean circumferential strain or the variance of circumferential strain.

**Table 3:** Mean and variance of mid-ventricular segmental circumferential strain (E_cc_) obtained using the conventional user-assisted and deep learning (DL)-based fully-automatic methods

	Segment 1	Segment 2	Segment 3	Segment 4	Segment 5	Segment 6
Mean E_cc_ (user-assisted)	− 0.13 ± 0.06	− 0.12 ± 0.05	− 0.15 ± 0.07	− 0.16 ± 0.09	− 0.15 ± 0.07	− 0.15 ± 0.06
Mean E_cc_ (automated DL)	− 0.13 ± 0.06	− 0.12 ± 0.06	− 0.15 ± 0.07	− 0.16 ± 0.09	− 0.16 ± 0.08	− 0.15 ± 0.06
Variance of E_cc_ (user-assisted)	1E–4 ± 1E–4	6E–4 ± 8E–4	1E−3 ± 2E−3	9E−4 ± 7E−4	5E−4 ± 5E−4	8E−4 ± 1E−3
Variance of E_cc_ (automated DL)	3E−4 ± 3E−4	6E−4 ± 5E−4	1E−3 ± 2E−3	1E−3 ± 1E−3	5E−4 ± 3E−4	8E−4 ± 1E−3

Segment 1: anteroseptal, segment 2: inferoseptal, segment 3: inferior, segment 4: inferolateral, segment 5: anterolateral, segment 6: anterior

## Discussion

We developed DL-based fully-automated methods for global and segmental strain analysis of short-axis DENSE CMR from a multicenter dataset. U-Nets were designed, trained and found to be effective for LV segmentation, identification of the anterior RV-LV insertion point, and phase unwrapping. Subsequent steps involving displacement and strain calculations were already automatic, thus, with the DL methods, the entire DENSE analysis pipeline for global and segmental strain is fully automated. Our studies validated the performance of each individual step, including segmentation, identification of the RV-LV insertion point, and phase unwrapping, and also validated the end-to-end performance of the entire pipeline by showing excellent correlation and agreement of whole-slice and segmental strain with well-established user-assisted semi-automatic methods.

Our 2D U-Net approach to LV segmentation was similar to other published methods for CMR, such as those used for cine CMR [19–22, 24, 26, 28], LGE [27], T_1_-weighted CMR [25] and phase contrast [23]. Three-dimensional convolutions may have advantages for segmentation of cine CMR data through time; however, they are less well studied for cine CMR than 2D and they will have their own unique challenges (such as the need for a constant number of cardiac phases, as one example). For cine CMR, to date most studies use a 2D model and achieve very good results [26, 28, 41]. Since 2D models work well and our DICE values are reasonably good using a 2D approach, our assessment was that a 2D U-Net was a reasonable choice. Also, our values for Hausdorff distance and MSD are similar to the mean contour distance of 1.14 mm and Hausdorff distance of 3.16 – 7.25 mm for myocardial segmentation reported by others [19], and to the average perpendicular distance of 1.1 ± 0.3 mm also reported by others [26]. We trained two separate U-Nets for epicardial and endocardial segmentation, although later we found that training one network for myocardial segmentation based on the proposed network architecture gives the same performance. The only difference was to define three classes of the blood pool, myocardium and background and to assign class weights of 3, 5 and 1, respectively, to overcome the imbalanced classes problem.

Our use of a semantic-segmentation U-Net and our data augmentation methods for phase unwrapping are new. For data augmentation, by applying prior segmentation and phase unwrapping methods, we had access to segmented and phase unwrapped data. Using simple manipulations of these data, as shown in Fig. 2, we were able to generate augmented pairs of wrapped and unwrapped images with new wrapping patterns, providing a very effective data augmentation strategy for training the phase-unwrapping U-Net. This strategy led to a more robust and successful CNN. In addition, the phase-unwrapping problem could potentially be treated using two different approaches. One approach would be to train a network to directly estimate the unwrapped phase from the potentially-wrapped input phase, i.e., treating the problem as a regression problem [42, 43]. Another approach, the one we took, is to estimate the integer number of wrap cycles at each pixel of the phase map by training a semantic-segmentation network to label each pixel according to its wrap class as defined in Table 1 [35, 44–46]. While both approaches are reasonable, we selected the semantic-segmentation approach because, by recognizing DENSE phase wrap patterns, we reasoned that the method may be effective even for low-SNR images; furthermore, the regression output does not apply any constraints to the output phase so that the network may yield unrealistic values. Our evaluation demonstrated the superiority of the semantic-segmentation phase-unwrapping network compared to path-following for low-SNR data. We did not directly compare the semantic-segmentation phase-unwrapping CNN to a CNN for direct phase estimation. Such a comparison could be performed in the future.

Prior work has demonstrated accurate analysis of DENSE images by segmenting the myocardium using just three manually-drawn contours instead of contouring all cardiac phases [47]. However, experience with this simplified method is limited to one site and a relatively small number of patients compared to methods that contour all cardiac phases, which have been used by many sites and applied to more patients. Also, the simple method relies on an accurate end-diastolic endocardial contour, which can be challenging to delineate because DENSE has poor blood-myocardium contrast at this particular cardiac phase. In addition, we had concerns that DL-based phase unwrapping may perform poorly using the suboptimal endocardial contours of the simplified method. For these reasons, we segmented all cardiac phases, and achieved good results. Lastly, we note that our DL methods provide a superset of the contours needed for the simplified method, so that a DL-based simplified method could be investigated.

While other strain imaging methods may provide reliable and reproducible global strain values and are well-suited to automatic DL-based analysis [20, 28, 29], cine DENSE has shown excellent reproducibility of segmental strain [7]. Our present results build upon these prior findings for segmental strain, and show excellent agreement of DL-based fully-automated segmental strain with user-assisted semi-automatically computed segmental strain. Interestingly, our limits of agreement for DL automatic vs. user-assisted segmental circumferential strain are better than those for DL vs. user-assisted analysis of myocardial-tagging-based global circumferential strain [29]. A potential explanation for the substantially better results for DENSE is that for tag analysis, DL is used to perform motion tracking, and even when trained using data from thousands of subjects, there is error in motion tracking [29]. In contrast, for DENSE, DL is used only for segmentation and phase unwrapping, but DL is not used for automatic motion estimation. For DENSE, during data acquisition displacement is encoded directly into the pixel phase, thus there is no need to learn motion estimation from image features. In essence, the motion estimation problem for DENSE is much simpler than for methods like tagging and feature tracking, and the demands for DL to accomplish full automation are much less.

This study focused on results for circumferential strain and not for radial strain. There are fewer pixels radially across the LV wall in short-axis images than circumferentially. For this reason, methods like DENSE and tagging are less accurate and reproducible for the estimation of radial strain compared to circumferential strain, and essentially all clinical applications of short-axis DENSE (and tagging) find that circumferential strain is diagnostically or prognostically useful, whereas radial strain does not perform as well. Accordingly, in our assessment, circumferential strain is emerging as an important biomarker in CMR, whereas radial strain is not.

Our study has limitations. We trained and evaluated our methods for short-axis cine DENSE data from multiple centers and different field strengths (1.5 T and 3 T), but from a single manufacturer. In the future, the networks may be trained using long-axis cine DENSE data to compute longitudinal strain and using data from other manufacturers if they provide the DENSE pulse sequence. Also, all the training data were manually contoured by just one expert reader. In the future we will have different readers perform contouring and we will retrain the networks so that they will be more generalized. Another limitation is that we trained the phase-unwrapping network to handle one cycle of phase wrap; however, occasionally we have observed DENSE images with two cycles of phase wrap. Future work can train the network for this case. It is worth mentioning that our data augmentation method for phase manipulation may be particularly useful for this case, as very few real datasets have two cycles of phase wrap. Moreover, in this study, we did not intentionally include training and testing datasets with artifacts due to respiratory motion or aliasing, or use images where the heart is not centered in the field of view. In future, we can add such datasets in order to develop more robust networks. Finally, although we obtained good results, our networks may be more robust if we further train them using more datasets from multiple sites and more heart-disease patients.

## Conclusions

The present study trained CNNs to perform LV segmentation, phase unwrapping, and identification of the anterior RV-LV insertion point for short-axis cine DENSE images, providing for fully-automatic global and segmental DENSE strain analysis with excellent agreement with conventional user-assisted methods. DL-based automatic strain analysis for DENSE may facilitate greater clinical use of DENSE for the assessment of global and segmental strain in patients with cardiac disease (Additional file 1, Additional file 2).

## Supplementary Information

**Additional file 1: Figure S1.** Examples showing the measurement of displacement SNR for a typical study (a) and for a stimulated case of low SNR (b). **Table S1.** Mean and standard deviation of radia strain for healthy volunteers and patients

**Additional file 2: Figure S1.** Bland–Altman plots for global (A) and segmental (B) radial strains at end systole of basal, mid-ventricular, and apical slices computed using the conventional user-assisted and the fully-automated DL methods. **Table S1.** Mean and standard deviation of radia strain for healthy subjects and patients

## Data Availability

The dataset used and analyzed during this study is available from the corresponding author on reasonable request.

## Abbreviations

CMR: Cardiovascular magnetic resonance
CNN: Convolutional neural network
DENSE: Displacement encoding with stimulated echoes
DL: Deep learning
E_cc_: Circumferential strain
ED: End-diastole
ES: End-systole
LGE: Late gadolinium enhancement
LV: Left ventricle/left ventricular
LVEF: Left ventricular ejection fraction
MSD: Mean surface distance
MSE: Mean squared error
NW: No wrap
ROI: Region of interest
RV: Right ventricle/right ventricular
SNR: Signal-to-noise ratio
