# Supplementary material for: Fully-automated global and segmental strain analysis of DENSE cardiovascular magnetic resonance using deep learning for segmentation and phase unwrapping
Source: J Cardiovasc Magn Reson. 2021 Mar 11;23:20. doi: 10.1186/s12968-021-00712-9 (PMC7949250; doi:10.1186/s12968-021-00712-9)
Supplement: Supplementary file 1 — Additional file 1: Figure S1. Examples showing the measurement of displacement SNR for a typical study (a) and for a stimulated case of low SNR (b). Table S1. Mean and standard deviation of radia strain for healthy volunteers and patients [file 12968_2021_712_MOESM1_ESM.pptx]

## Slide 1
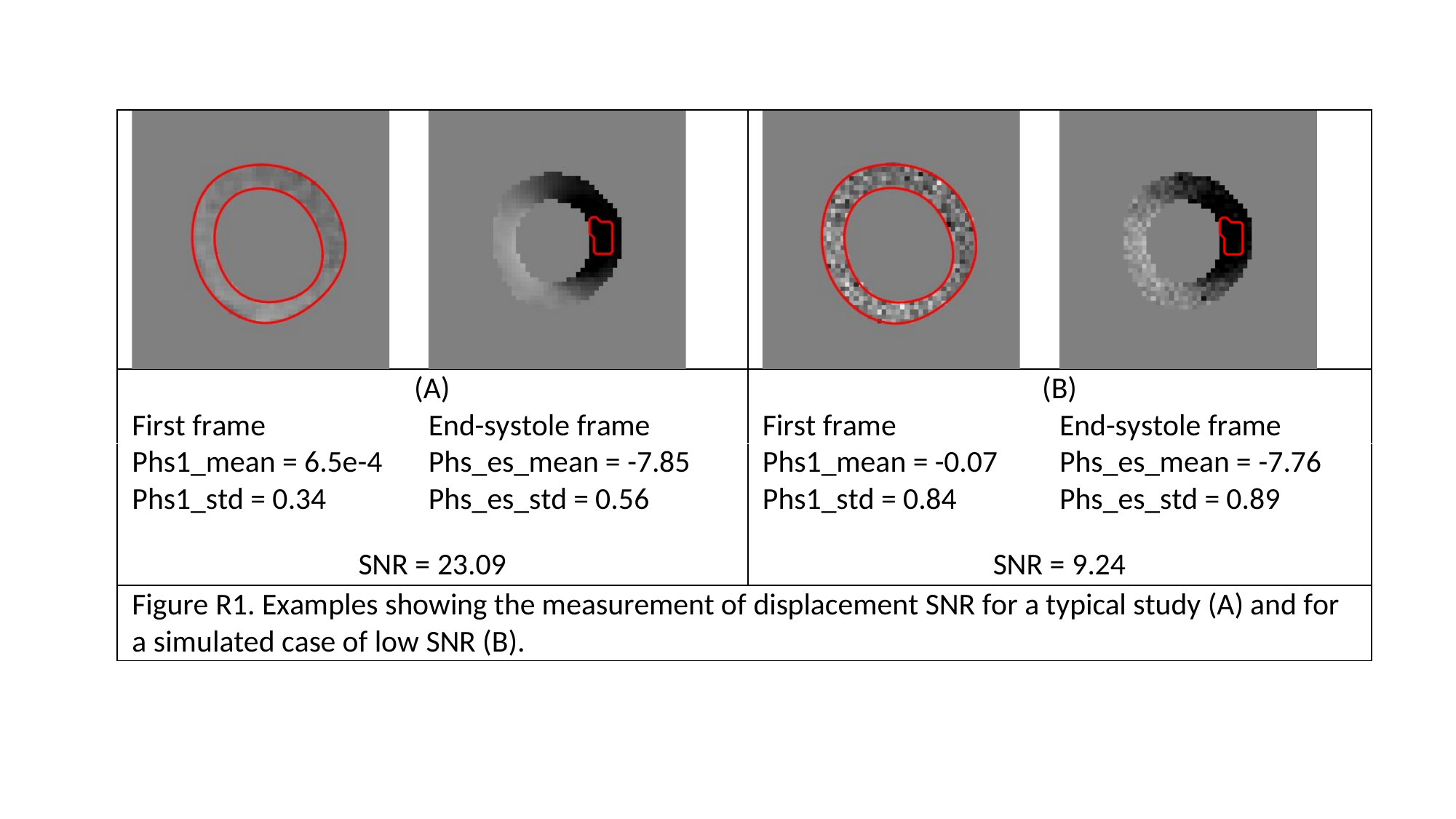

## Slide 2
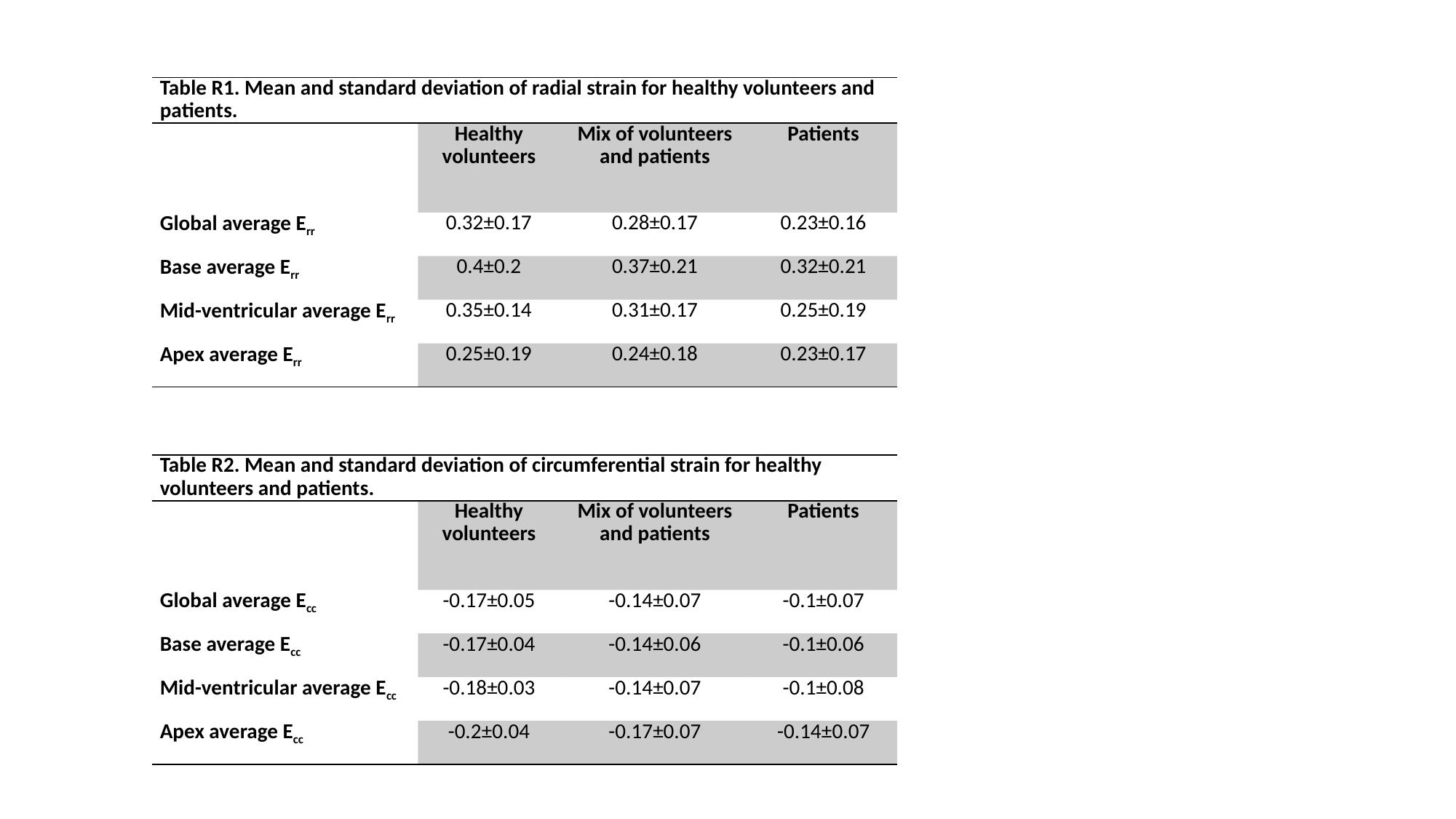

| Table R1. Mean and standard deviation of radial strain for healthy volunteers and patients. | | | |
| --- | --- | --- | --- |
| | Healthy volunteers | Mix of volunteers and patients | Patients |
| Global average Err | 0.32±0.17 | 0.28±0.17 | 0.23±0.16 |
| Base average Err | 0.4±0.2 | 0.37±0.21 | 0.32±0.21 |
| Mid-ventricular average Err | 0.35±0.14 | 0.31±0.17 | 0.25±0.19 |
| Apex average Err | 0.25±0.19 | 0.24±0.18 | 0.23±0.17 |
| Table R2. Mean and standard deviation of circumferential strain for healthy volunteers and patients. | | | |
| --- | --- | --- | --- |
| | Healthy volunteers | Mix of volunteers and patients | Patients |
| Global average Ecc | -0.17±0.05 | -0.14±0.07 | -0.1±0.07 |
| Base average Ecc | -0.17±0.04 | -0.14±0.06 | -0.1±0.06 |
| Mid-ventricular average Ecc | -0.18±0.03 | -0.14±0.07 | -0.1±0.08 |
| Apex average Ecc | -0.2±0.04 | -0.17±0.07 | -0.14±0.07 |
