# Supplementary material for: Fully-automated global and segmental strain analysis of DENSE cardiovascular magnetic resonance using deep learning for segmentation and phase unwrapping
Source: J Cardiovasc Magn Reson. 2021 Mar 11;23:20. doi: 10.1186/s12968-021-00712-9 (PMC7949250; doi:10.1186/s12968-021-00712-9)
Supplement: Supplementary file 2 — Additional file 2: Figure S1. Bland–Altman plots for global (A) and segmental (B) radial strains at end systole of basal, mid-ventricular, and apical slices computed using the conventional user-assisted and the fully-automated DL methods. Table S1. Mean and standard deviation of radia strain for healthy subjects and patients [file 12968_2021_712_MOESM2_ESM.pptx]

## Slide 1
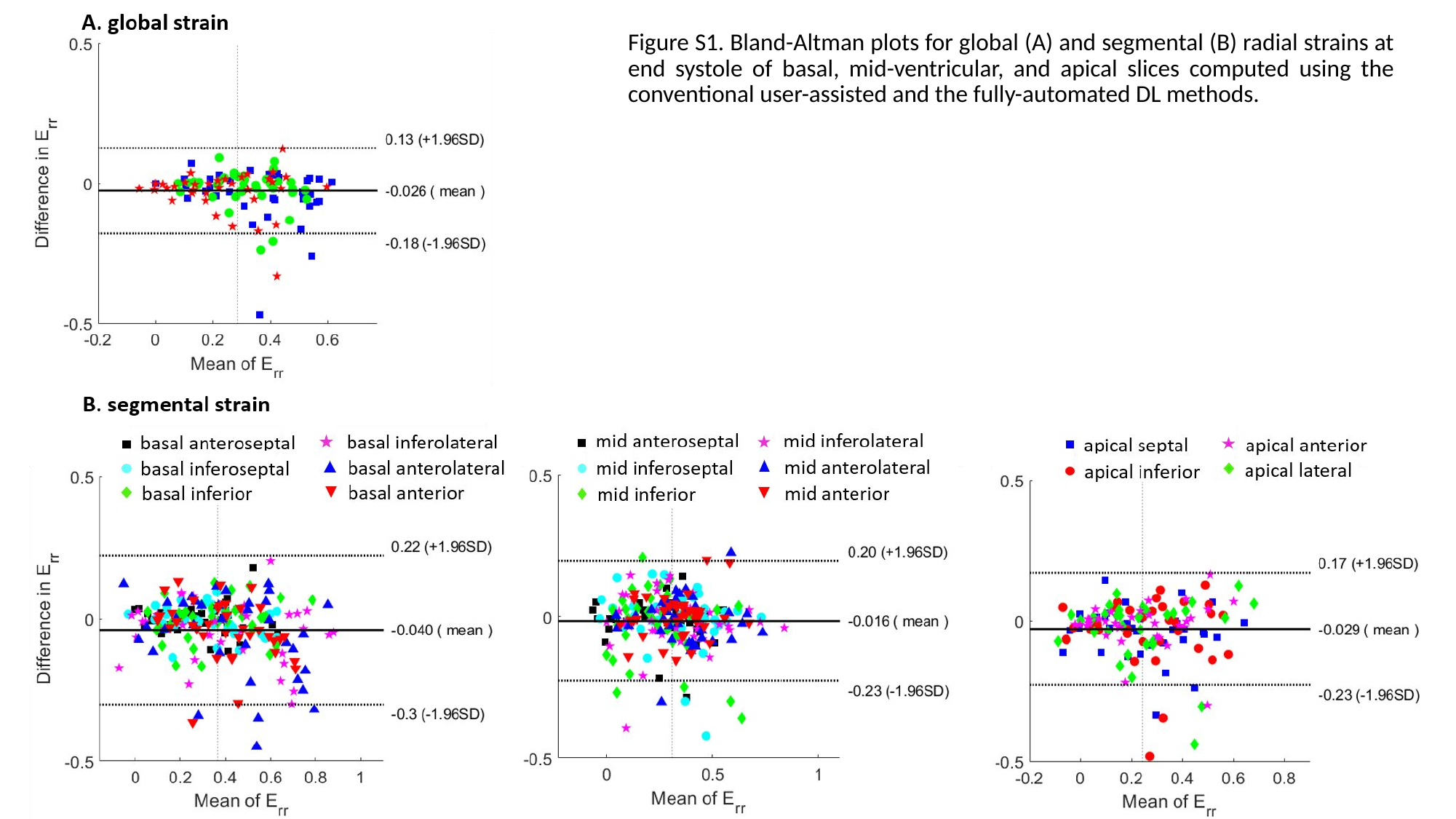

| Figure S1. Bland-Altman plots for global (A) and segmental (B) radial strains at end systole of basal, mid-ventricular, and apical slices computed using the conventional user-assisted and the fully-automated DL methods. |
| --- |

## Slide 2
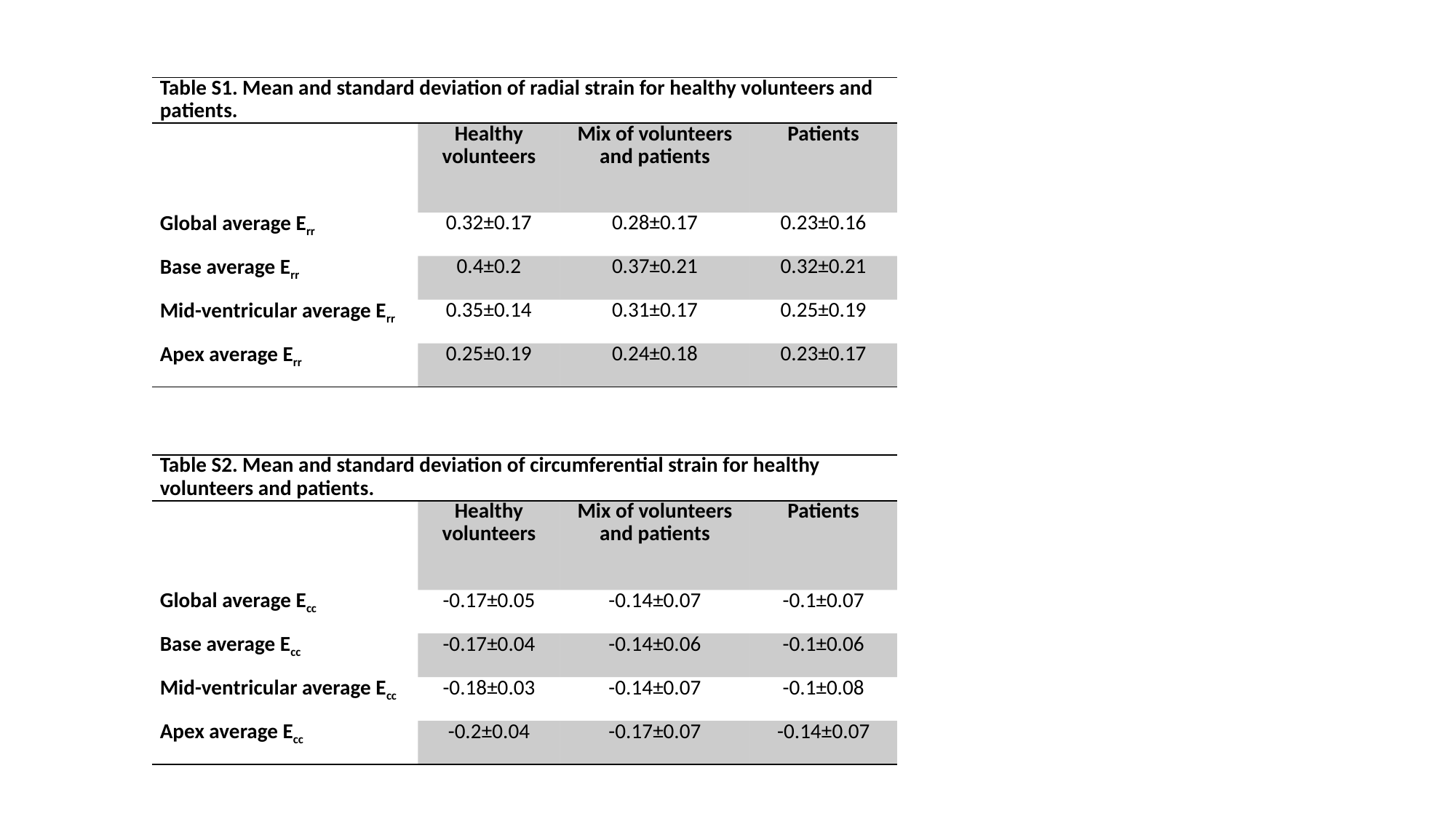

| Table S1. Mean and standard deviation of radial strain for healthy volunteers and patients. | | | |
| --- | --- | --- | --- |
| | Healthy volunteers | Mix of volunteers and patients | Patients |
| Global average Err | 0.32±0.17 | 0.28±0.17 | 0.23±0.16 |
| Base average Err | 0.4±0.2 | 0.37±0.21 | 0.32±0.21 |
| Mid-ventricular average Err | 0.35±0.14 | 0.31±0.17 | 0.25±0.19 |
| Apex average Err | 0.25±0.19 | 0.24±0.18 | 0.23±0.17 |
| Table S2. Mean and standard deviation of circumferential strain for healthy volunteers and patients. | | | |
| --- | --- | --- | --- |
| | Healthy volunteers | Mix of volunteers and patients | Patients |
| Global average Ecc | -0.17±0.05 | -0.14±0.07 | -0.1±0.07 |
| Base average Ecc | -0.17±0.04 | -0.14±0.06 | -0.1±0.06 |
| Mid-ventricular average Ecc | -0.18±0.03 | -0.14±0.07 | -0.1±0.08 |
| Apex average Ecc | -0.2±0.04 | -0.17±0.07 | -0.14±0.07 |
